# Cellular and Network Contributions to Excitability of Layer 5 Neocortical Pyramidal Neurons in the Rat

**DOI:** 10.1371/journal.pone.0001209

**Published:** 2007-11-21

**Authors:** Dan Bar-Yehuda, Alon Korngreen

**Affiliations:** 1 The Mina and Everard Faculty of Life Sciences, Bar-Ilan University, Ramat-Gan, Israel; 2 Leslie and Susan Gonda Multidisciplinary Brain Research Center, Bar-Ilan University, Ramat-Gan, Israel; Vrije Universiteit Amsterdam, Netherlands

## Abstract

There is a considerable gap between investigating the dynamics of single neurons and the computational aspects of neural networks. A growing number of studies have attempted to overcome this gap using the excitation in brain slices elicited by various chemical manipulations of the bath solution. However, there has been no quantitative study on the effects of these manipulations on the cellular and network factors controlling excitability. Using the whole-cell configuration of the patch-clamp technique we recorded the membrane potential from the soma of layer 5 pyramidal neurons in acute brain slices from the somatosensory cortex of young rats at 22°C and 35°C. Using blockers of synaptic transmission, we show distinct changes in cellular properties following modification of the ionic composition of the artificial cerebrospinal fluid (ACSF). Thus both cellular and network changes may contribute to the observed effects of slice excitation solutions on the physiology of single neurons. Furthermore, our data suggest that the difference in the ionic composition of current standard ACSF from that of CSF measured *in vivo* cause ACSF to depress network activity in acute brain slices. This may affect outcomes of experiments investigating biophysical and physiological properties of neurons in such preparations. Our results strongly advocate the necessity of redesigning experiments routinely carried out in the quiescent acute brain slice preparation.

## Introduction

*In vitro* experiments, mostly in acute slice preparations, allow repeated recording from visually identified neurons and micro-networks of neurons [1]–[5]. However, background activity in brain slices is low. While this is beneficial for many valuable projects striving to isolate properties of single neurons, the low excitability leads primarily to a higher membrane resistance (R_m_) than observed *in vivo*. This high R_m_ increases the membrane time constant (τ) and the passive space constant (λ). Therefore, the integration of synaptic input in slice preparations may not truly reflect the conditions found *in vivo* [6]–[9]. This problem raises fundamental questions [10]: To what extent can results obtained *in vitro* tell us about synaptic integration *in vivo*? Is scaling membrane properties in cellular models obtained *in vitro* enough to reliably simulate the *in vivo* situation?

Attempts have been made to simulate the response of a single neuron to network activity *in vitro* by injecting white or “colourful” noise and by a more sophisticated modification of the patch-clamp technique called dynamic-clamp [11]–[20]. Nevertheless, while providing many insights, the dynamic-clamp technique still does not fully reflect conditions *in vivo*. Application of the dynamic-clamp requires current injection via a whole-cell electrode and therefore simulates the activation of a point conductance and not an overall change of synaptic input. Thus, changes to R_m_ are not global as predicted for the *in vivo* situation.

To overcome the shortcomings of *in vitro* preparations and to identify modulations of synaptic input, chemical modification of the bath solution has been used to increase background synaptic activity in brain slices [21]–[23]. This approach allows the production of synchronous activity of many neurons in the slice, thus mimicking synaptic integration during intense synaptic activity. However, it is not clear how the various slice excitation methods modulate the intrinsic properties of the individual neurons. Our working hypothesis stated that slice excitation media will cause changes to both cellular and network parameters. To investigate this hypothesis we performed whole-cell recordings from layer 5 pyramidal neurons during slice excitation. We report that modulation of slice excitation level by modifying the ionic composition of the bath solution modifies both cellular and network excitability of L5 pyramidal neurons.

## Methods

### Slice preparation

Sagittal slices of 300 µm were prepared from the cortex of 13–15 days old Wistar rats killed by rapid decapitation following shallow anesthesia with isoflurane, in accordance with the guidelines of the Bar-Ilan University animal welfare committee, using previously described techniques [1]. Following 30 minutes incubation at 35°C the slices were maintained in a submersion-type chamber at room temperature for the remainder of the day. Slices were perfused throughout the experiment with an oxygenated artificial cerebrospinal fluid (ACSF) containing: (mM) 125 NaCl, 15 NaHCO_3_, 2.5 KCl, 1.25 NaH_2_PO_4_, 1 MgCl_2_, 2 CaCl_2_, 25 glucose (pH 7.4 with 5% CO_2_, 310 mosmol kg^−1^). Modifications of this solution are noted in Table 1. Experiments reported here were mostly carried out at room temperature (20–22°C) and selected experiments were repeated at 35°C. Pyramidal neurons from layer 5 in the somatosensory cortex were visually identified using infrared differential interference contrast (IR-DIC) videomicroscopy [1].

**Table 1:** Composition of tested extracellular solutions.

	ACSF	ACSF_1_	ACSF_2_	ACSF_3_
KCl (mM)	2.5	3.5	6.25	2.5
CaCl_2_ (mM)	2	1.2	1.5	2
MgCl_2_ (mM)	1	1	0.5	1
NMDA (µM)	0	0	0	x
AMPA (µM)	0	0	0	0.1x

The table lists only those components of ACSF modified during the experiments. The remaining ACSF ingredients are listed in Methods. ACSF_1_ was taken from Sanchez-Vives *et al* [21]. ACSF_2_ was taken from Silberberg *et al* [22]. The concentration of AMPA was 10 times smaller (0.1x) than the concentration of NMDA (x) in all experiments.

### Solutions and Drugs

The standard pipette solution contained (mM): 125 K-gluconate, 20 KCl, 10 HEPES, 4 MgATP, 10 Na_2_-phosphocreatin, 0.5 EGTA, 0.3 GTP and 0.5% (w/w) biocytin (Sigma) (pH 7.2 with KOH, 312 mosmol kg^−1^). D(-)-2-amino-5-phosphonopentanoic acid (AP-5, Tocris, Bristol, UK), 6-cyano-7-nitroquinoxaline-2,3-dione (CNQX, Tocris) and bicuculine (Tocris) were stored as stock solutions in doubly distilled water. Glutamate receptor agonists alpha-amino-3-hydroxy-5-methyl-4-isoxazolepropionic acid (AMPA, Sigma) and N-methyl-D-aspartic acid (NMDA, Sigma) were both stored as stock solution in doubly distilled water and added to the bath solution shortly before the experiment. The reference electrode was an Ag-AgCl pellet placed in the bath. The 11 mV liquid junction potential measured under the ionic conditions reported here was not corrected for. This liquid junction potential was not altered by the ionic modifications to the ACSF reported in Table 1.

### Whole-cell recordings

Whole-cell recordings were performed from the soma of layer 5 pyramidal neurons using a Multiclamp-700B or Axopatch-200B amplifiers (Axon Instruments, Foster City, CA). Voltage was filtered at 10 kHz and sampled at 50 or 20 kHz using AxoClamp9 (Axon Instruments, Foster City, CA), digitized by a Digidata-1320 interface (Axon Instruments, Foster City, CA), and stored on the hard disk of a personal computer. Patch pipettes (4–7 MΩ) were pulled from thick walled borosilicate glass capillaries (2.0 mm outer diameter, 0.5 mm wall thickness, Hilgenberg, Malsfeld, Germany).

### Analysis

All off-line data analysis was carried out with IgorPro 5.0 (WaveMetrics, Lake Oswego, USA) on a personal computer. Action potential (AP) threshold was extracted by numerically calculating the second derivative of the membrane potential as a function of time. The threshold was determined to be the point in which the second derivative exceeded 50% of its maximal value (see Fig. S1A for a graphical display of this procedure). Action potential amplitude was measured from threshold to the peak of the AP. Input resistance (R_in_) was measured by injecting several hyperpolarizing current steps, measuring the maximal voltage deflection from the resting membrane potential, subtracting the resting membrane potential, and calculating R_in_ by linear regression (see Fig. S1B for a graphical display of this procedure). During these measurements no holding current was injected via the patch-pipette to avoid changes to R_in_ resulting from voltage-dependent modulation of ion channels. Current-Frequency curves were generated by injecting a series of 1 second depolarizing current steps via the patch pipette. The mean firing frequency was calculated from the last 600 ms of each recorded trace where the firing rate reached a steady state (see Fig. S1C for a graphical display of this procedure). Current-frequency curves were analyzed using exponential curve fitting. The current required to reach 63% of the maximal firing rate was extracted from each fit and used for comparison with other experimental conditions. Instantaneous firing frequency histograms were compiled by extracting the inter spike intervals from 6 minute long periods of stable activity. The firing frequency histogram was fitted to Gaussian curves in order to extract the peaks of the histogram components. All point histograms of subthreshold membrane potential were compiled from 120 seconds long recording segments that did not display AP firing. During slice excitation shorter homogeneous segments without APs were joined together to reach that length before the generation of the histogram. Experimental results were consistently observed in cells from six or more animals, hence all the results for a particular experiment were pooled and displayed as means±S.E.M save for the firing rate histograms where histogram properties were reported as means±S.D.; groups were compared with Student's *t* test.

## Results

The aim of the current study was to quantitatively compare the effectiveness of slice excitation protocols. We tested three protocols that have been previously reported to induce network activity in acute brain slices. Two protocols use modified ionic composition of the ACSF to excite the neural network in the slice, while the third uses bath application of excitatory neurotransmitters. The first protocol we tested was modified from Sanchez-Vives *et al.* [21] by increasing the [K^+^] in the ACSF from 2.5 to 3.5 mM and decreasing [Ca^2+^] in the ACSF from 2 to 1.2 mM (Table 1). This modified ACSF will be referred to as ACSF_1_. The second protocol tested was based on that of Silberberg *et al.* [22] and entailed substantially larger modifications to the ACSF than ACSF_1_. The extracellular [K^+^] was increased from 2.5 to 6.25 mM, [Ca^2+^] was decreased from 2 to 1.5 mM, and [Mg^2+^] was decreased from 1 to 0.5 mM (Table 1). This solution will be referred to as ACSF_2_. The third protocol used for slice excitation did not employ ionic modifications of the ACSF. Instead various concentrations of AMPA and NMDA were added to the ACSF as shown in table 1. In all experiments a constant ratio was maintained between AMPA and NMDA, NMDA concentration being 10 times that of AMPA. This excitatory medium will be referred to as ACSF_3_. Variants of this excitation solution have been used previously [24]–[28]. However, to the best of our knowledge, this is the first time that brain slices were excited using the specific combination of NMDA and NMPA reported in Table 1.

**Figure 1:**
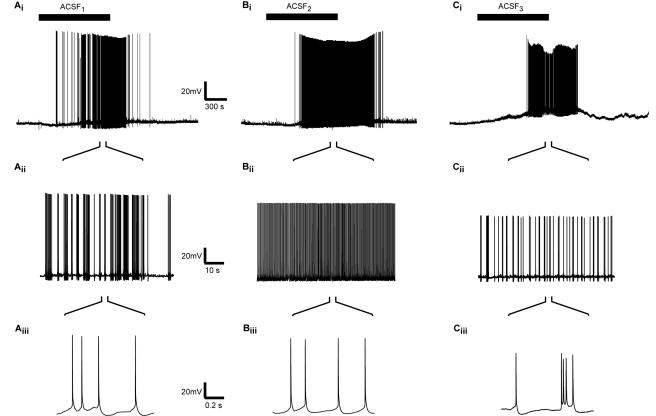
Effect of excitatory media on the firing of L5 pyramidal neurons. 45 minutes of whole-cell recordings in the control condition, application of modified ACSF (marked by solid bar above A_i_, B_i_ and C_i_) and washout. Columns A, B and C show, respectively, application of ACSF_1_, ACSF_2_ and ACSF_3_ with 8 µM NMDA and 0.8 µM AMPA (see table 1). To observe the spiking events, A_ii_, B_ii_ and C_ii_ in the middle row are enlarged from the application period of A_i_, B_i_ and C_i_, with further enlargement given in A_iii_, B_iii_ and C_iii_.

Replacing the ACSF in the bath with any of these three excitatory solutions generated repeated action potential firing in L5 pyramidal neurons with a delay of at least 12 minutes (Fig 1). This delay was probably due to the time required for complete substitution of the bath solution by the perfusion system, the permeation of the excitatory solution through the brain slice and the activation of enough neurons to enable reverberating activity in the network. Application of ACSF_1_ generated a slow increase in the firing rate characterized by episodic bursts of APs (Fig. 1A). Conversely, the rapid elevation in the AP rate generated by application of ACSF_2_ appeared to consist of a regular firing (Fig. 1B). Application of ACSF_3_ containing 8 µM NMDA and 0.8 µM AMPA produced an activation pattern similar to that generated by ACSF_1_ (Fig. 1C).

**Figure 2:**
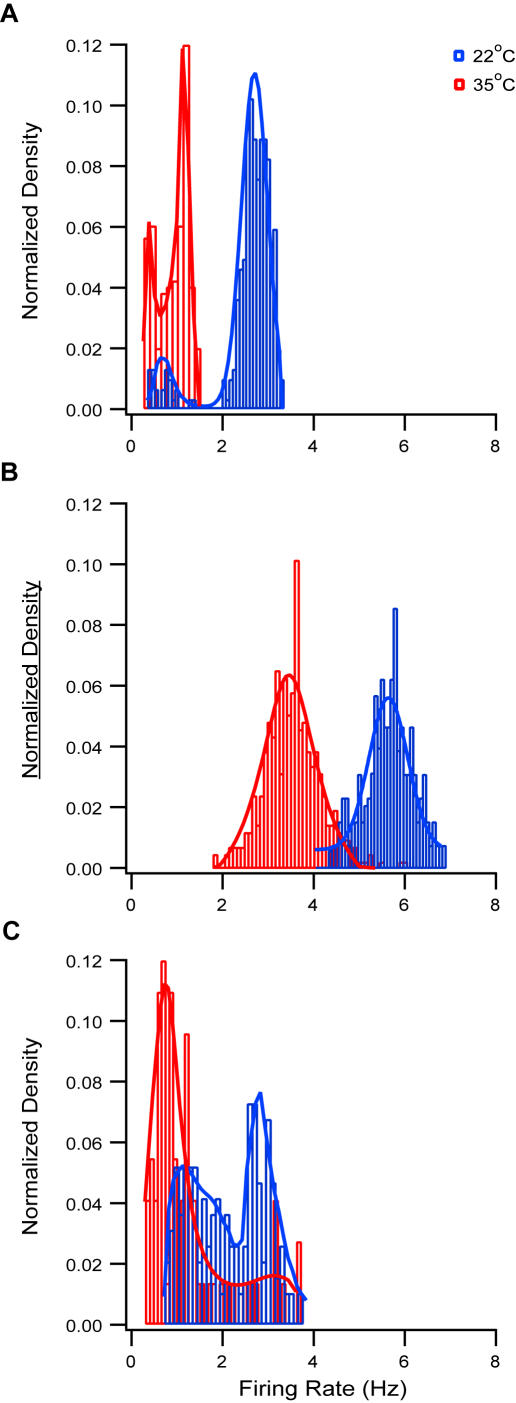
Instantaneous firing rate histograms in excited brain slices. Room temperature of 22°C (blue) and physiological temperature of 35°C (red). The instantaneous firing rate histograms were calculated from continuous recording of the membrane potential following the application of ACSF_1_ (A), ACSF_2_ (B) and ACSF_3_ with 8 µM NMDA and 0.8 µM AMPA (C). The histograms were fitted with a sum of two Gaussian distributions in A and C. The histograms in B were fitted only with one Gaussian distribution. In each subfigure the histograms were compiled from a single neuron.

The firing patterns observed visually were echoed by the instantaneous firing rate histograms. Representative instantaneous firing frequency histograms derived following slice excitation are displayed in figure 2. Application of ACSF_1_ at room temperature generated a histogram with two distinct peaks. A major peak at 2.7±0.3 Hz constituting 92±4% of the surface of the histogram (n = 5) and a minor peak at 0.7±0.2 Hz constituting 8±4% of the surface of the histogram (Fig. 2A). When the experiment was carried out at 35°C both peaks of the histogram were shifted to lower frequencies. The major peak shifted to 1.2±0.1 Hz constituting 59±21% of the surface of the histogram (n = 7) and the minor peak to 0.4±0.1 Hz constituting 41±21% of the surface of the histogram (Fig. 2A). Conversely, the firing frequency histograms displayed only one peak when the slices were excited using ACSF_2_ (Fig. 2B). At room temperature the peak of the histogram was at 5.3±0.4 Hz (n = 8) which shifted to 3.5±0.6 Hz when the experiments were carried out at 35°C. Similarly to ACSF_1_ application of ACSF_3_ containing 8 µM NMDA and 0.8 µM AMPA also generated firing histograms with two distinct peaks (Fig. 2C). At room temperature the two peaks were at 3.2±0.3 Hz (48±4% contribution, n = 6) and 1.3±0.5 Hz (52±4% contribution, n = 6) whereas at 35°C the peaks were at 3±0.3 Hz (74±12% contribution, n = 6) and 0.7±0.3 Hz (26±12% contribution, n = 6).

**Figure 3:**
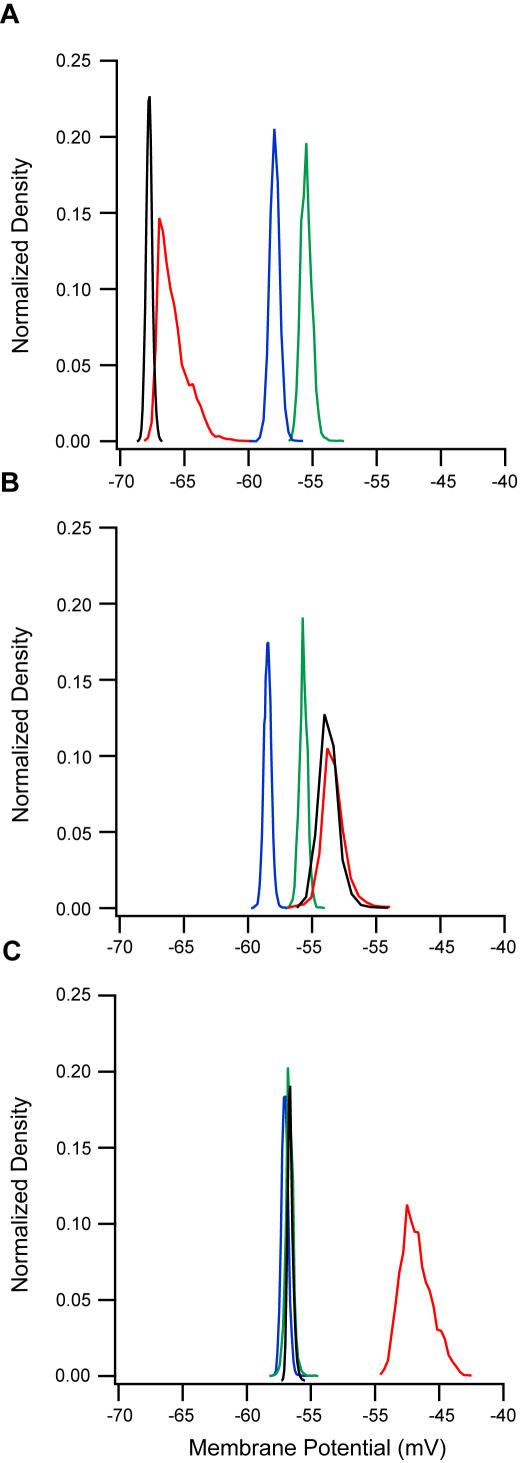
All-point histograms of subthreshold membrane potential in excited brain slices. Control (blue), application of modified ACSF (red), after adding synaptic blockers (black), and washout (green). A, B and C show, respectively, application of ACSF_1_, ACSF_2_ and ACSF_3_ with 8 µM NMDA and 0.8 µM AMPA (see table 1). In each subfigure the histograms were compiled from a single neuron.

To determine what part of the activation of L5 pyramidal neurons was due to the direct effect of the excitatory solutions on the cells and what was due to network activity, synaptic transmission was blocked during slice excitation. In order to block synaptic transmission, 50 µM AP-5, 15 µM CNQX and 20 µM bicuculine were added to the excitatory solution after firing appeared to reach a steady-state. The results are given in Figure 3, which shows recordings of the subthreshold membrane potential transformed into all-point-histograms. Following the application of ACSF_1_ the membrane potential histogram broadened (Fig. 3A), as would be expected from an increase in background synaptic activity [7]–[9], [29]. Surprisingly, the histogram was also shifted to lower membrane potential values (Fig. 3A). Blocking synaptic transmission with AP-5, CNQX and bicuculine reduced the width of the membrane potential histogram. However, the histogram remained shifted to membrane potential values that were more negative than the control histogram (Fig. 3A). Washout of ACSF_1_ with ACSF shifted the histogram back to the control values for membrane potential.

Performing the same experiment with ACSF_2_ as the excitatory solution produced a different pattern of activity at subthreshold potentials (Fig. 3B). The membrane potential histogram broadened and was shifted to more positive potentials. Blocking synaptic transmission did not change the width or position of the histogram (Fig. 3B). In four out of six experiments, the neurons continued to fire in the presence of synaptic blockers. This indicates that much of the effect of ACSF_2_ results from cellular and not network excitability. Washing out the ACSF_2_ reduced the width of the histogram and shifted it closer to control values (Fig. 3B). Exciting the brain slice with ACSF_3_ containing 8 µM NMDA and 0.8 µM AMPA generated a broadening and a shift to more positive potentials of the membrane potential histogram (Fig. 3C). This effect was completely blocked by blockers of synaptic transmission and was completely reversible following washout (Fig. 3C).

**Figure 4:**
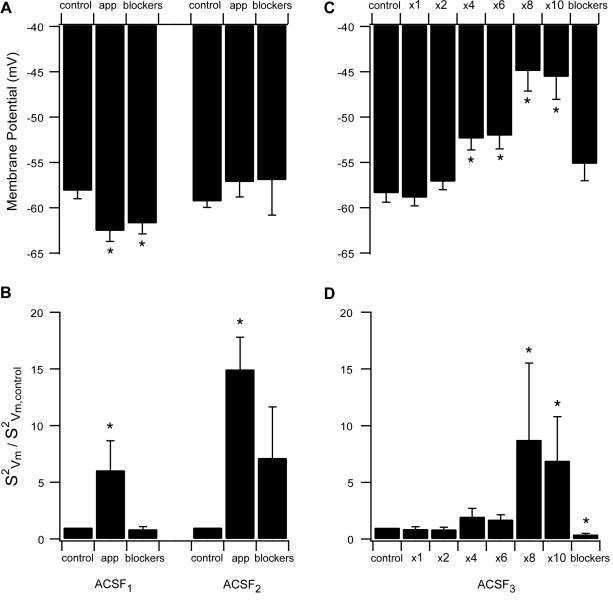
Effect of slice excitation on average membrane potential and variance. Membrane potential and membrane potential variance were measured under control conditions, application of modified ACSF and addition of synaptic blockers. The values of the membrane potential variance are displayed normalized to their control values. A, average membrane potential with ACSF_1_ (left) and ACSF_2_ (right). B, membrane potential variance with ACSF_1_ (left) and ACSF_2_ (right). C, average membrane potential with various concentrations of ACSF_3_. D, membrane potential variance with various concentrations of ACSF_3_.

To quantify the effects depicted in Figure 3 we calculated the mean and variance of the subthreshold membrane potential fluctuations in several such experiments (Fig. 4). Application of ACSF_1_ at room temperature generated a significant hyperpolarization in the membrane potential (Fig. 4A, n = 11, p<0.05, paired t-test) that was also sustained after synaptic transmission was blocked (n = 10, p<0.05 paired t-test), confirming the shift of the all-point histogram. Interestingly, application of ACSF_1_ at 35°C generated a significant depolarization in the membrane potential (Fig. S2A). The variance of the membrane potential increased significantly after application of ACSF_1_ (Fig. 4B, n = 11, p<0.05 paired t-test) but showed no statistically significant difference from the control values after adding synaptic blockers (n = 10, p>0.5 paired t-test). Application of ACSF_2_ also resulted in similar trends in the average variance of the membrane potential to those in Fig. 2 (Fig. 4B, n = 8, p<0.01 paired t-test). The average membrane potential was more depolarized in relation to control values, though not significantly so (Fig. 4A, n = 8, p = 0.15 paired t-test). Similar changes were observed at 35°C (Fig. S2A and S2B).

Application of ACSF_3_ with various concentrations of AMPA and NMDA generated a progressive change in the membrane potential (Fig. 4C). Only NMDA concentration of 4 µM together with the corresponding 0.4 µM AMPA concentration generated a significant depolarization compared to control level (n = 8, p<0.01, paired t-test). However, this progressive depolarization was linearly correlated to the neurotransmitter concentrations applied (R = 0.75, p<0.05). Note that with these combinations of neurotransmitter concentrations the membrane potential of L5 pyramidal neurons displayed only changes in subthreshold activity without generating action potentials. Spontaneous action potentials were observed when the NMDA concentration was increased to 8 µM (with the corresponding AMPA concentration of 0.8 µM, see also Fig. 1). In additional to generation of action potentials, we also observed a considerable increase in the variance of the subthreshold membrane potential (Fig. 4D, n = 7, p<0.01, paired t-test). Similar changes were observed at 35°C (Fig. S2A and 2B).

**Figure 5:**
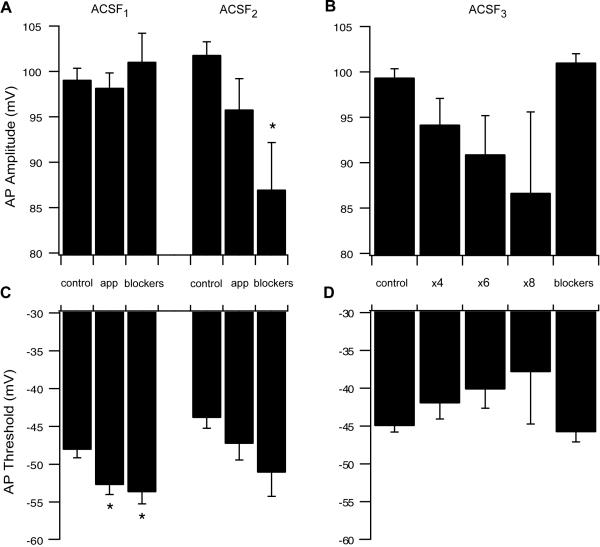
Effect of slice excitation on action potential amplitude and threshold. AP amplitude and threshold were measured under control conditions, ACSF application and addition of synaptic blockers. A, AP amplitude with ACSF_1_ (left) and ACSF_2_ (right). B, AP amplitude with various concentrations of ACSF_3_. C, AP threshold with ACSF_1_ (left) and ACSF_2_ (right). D, AP threshold with various concentrations of ACSF_3_.

Next we quantified several other parameters that convey information on the various passive and active properties of the neurons. The action potential amplitude was not significantly affected by either ACSF_1_ or ACSF_2_. The only significant change was observed in ACSF_2_ during application of synaptic blockers (Fig. 5A, p<0.05, n = 6). Increasing the concentrations of AMPA and NMDA in the ACSF_3_ solution decreased the AP amplitude in a concentration- dependent manner that was not, however, statistically significant (Fig. 5B). This is probably due to the AP amplitude varying greatly during application of ASCF_3_ solution, as can be seen in Figure 1Ci. The AP threshold was significantly modified only by ACSF_1_, which hyperpolarized it from -48.1+0.95 mV (n = 25) under control conditions to −52.75+1.15 mV (Fig. 5C, n = 21, p = 0.013 paired t-test). As with the AP amplitude, increasing the concentration of AMPA and NMDA in the bath medium generated a concentration-dependent depolarizing drift in the AP threshold (Fig. 5D) that was not statistically significant. Similar changes were observed at 35°C (Fig. S2C and 2D).

**Figure 6:**
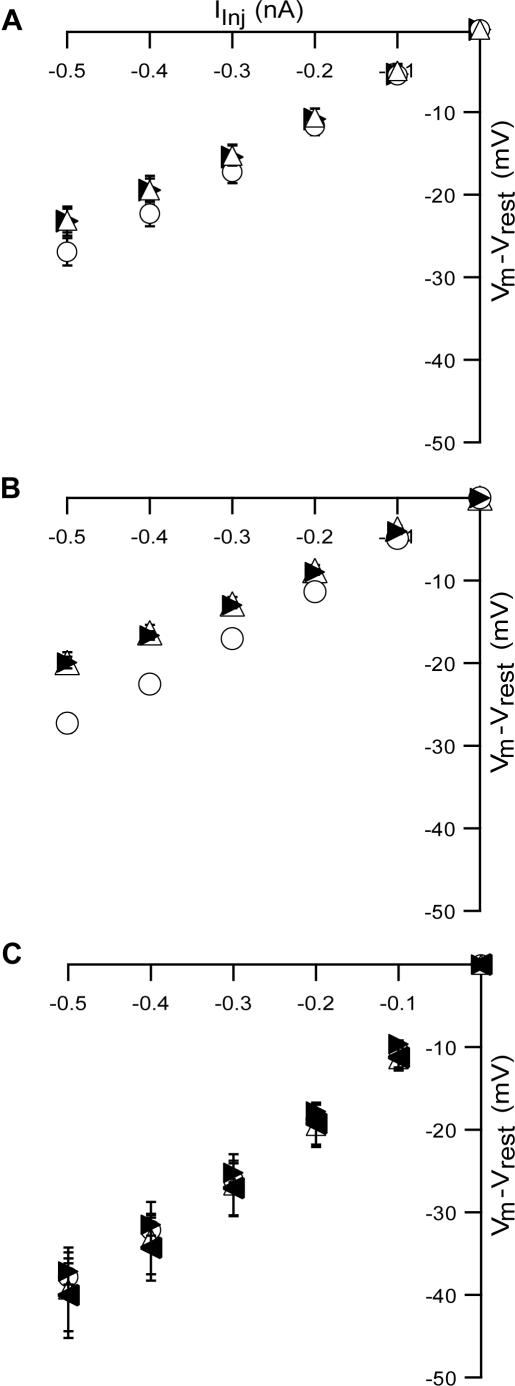
Modified ionic composition of ACSF changes R_in_ of L5 pyramidal neurons. Input resistance (R_in_) was extracted by a linear fit. A and B, data taken in the control condition (open circles), application of modified ACSF (open triangles) and application of modified ACSF with synaptic blockers (filled rightward triangles). A, voltage-current curves with ACSF_1_. B, voltage-current curves with ACSF_2_. C, voltage-current curves with ACSF_3_ in which the applications are generalized to ACSF_3_ with 4 µM NMDA and 0.4 µM AMPA (open triangles) ACSF_3_ with 8 µM NMDA and 0.8 µM AMPA (filled leftward triangles).

For a first order approximation of the changes in the passive properties of L5 pyramidal neurons with addition of the excitatory solutions, we constructed current-voltage relationships of the neurons using hyperpolarizing current pulses. As could have been predicted, changing the bath solution to ACSF_1_ significantly reduced R_in_ from the control value of 55±13 MΩ to 48±12 MΩ (Fig. 6A, n = 11, p<0.01, paired t-test). R_in_ did not return to the control value after the addition of synaptic blockers to ACSF_1_ (49±14 MΩ, n = 11, p<0.01, paired t-test). A larger decrease in R_in_, from 55±8 MΩ to 41±9 MΩ, occurred after application of ACSF_2_ (Fig. 6B, n = 6, p<0.01, paired t-test). As with ACSF_1_ R_in_ did not recover to control values after addition of synaptic blockers (Fig. 6B, 41±4 MΩ, n = 6, p<0.05, paired t-test). None of the neurotransmitter combinations tested (1, 2, 4, 6, 8, 10 µM NMDA and the corresponding 0.1, 0.2, 0.4, 0.6, 0.8, 1 µM AMPA) caused any significant change in R_in_ (Fig. 6C). Similar changes were observed at 35°C (Fig. S3A–S3C).

**Figure 7:**
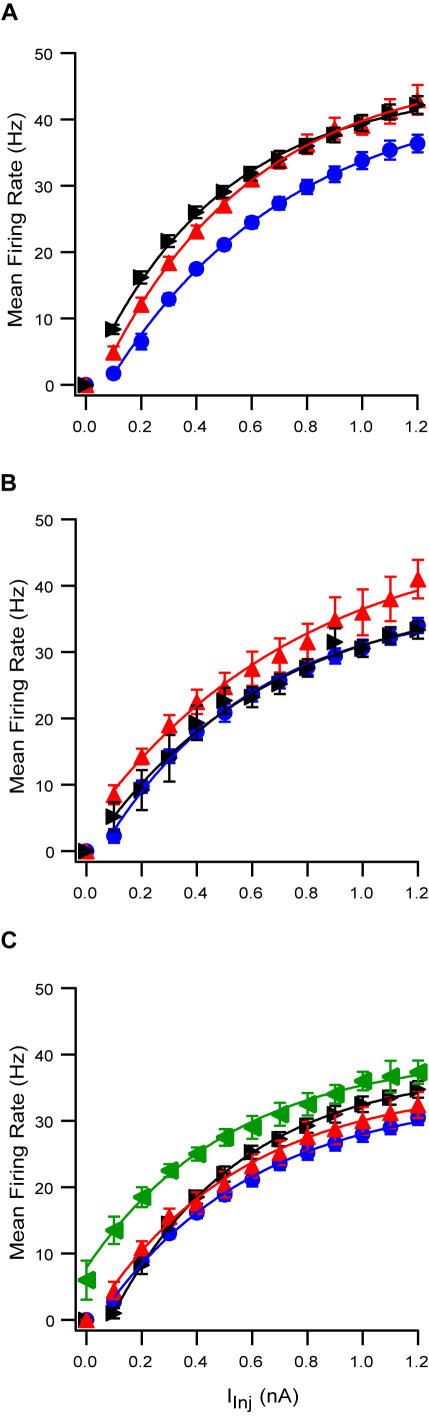
Modified ionic composition of ACSF changes response of L5 pyramidal neurons to injected current. Lines plotted by exponential fitting to curves from AP frequency vs. injected current. A and B, control (blue circles), application of modified ACSF (red upward triangle) and addition of synaptic blockers (black triangle). A, ACSF_1_; B, ACSF_2_; C, ACSF_3_ with 6 µM NMDA and 0.6 µM AMPA (red triangles) and ACSF_3_ with 8 µM NMDA and 0.8 µM AMPA (green triangles).

Finally, we generated current-frequency curves (F-I) to analyze the firing of L5 pyramidal neurons in response to depolarizing current pulses in the various excitation solutions (Fig. 7). Under control conditions the current required to induce a firing rate of 63% of the maximal firing rate was 0.65±0.06 nA (n = 20). Following application of ACSF_1_ the F-I shifted to lower current values such that the current required to reach 63% of the maximal firing frequency fell significantly to 0.54±0.06 nA (Fig. 7A, n = 18, p<0.01, t-test, and see Fig. S3D for the F-I curve obtained at 35°C). The F-I curve remained shifted to lower current values after the application of synaptic transmission blockers (0.47±0.04 nA, n = 11). Conversely, application of ACSF_2_ generated a shift in the F-I curve to higher current values (Fig. 7B, and see Fig. S3E for the F-I curve obtained at 35°C). The current required to reach 63% of the maximal firing frequency increased from 0.53±0.06 nA to 0.76±0.18 nA (n = 7, p<0.02, paired t-test). Application of ACSF_3_ resulted in an apparent shift in the F-I curve (Fig. 7C, and see Fig. S3F for the F-I curve obtained at 35°C). However, since the maximal firing rate increased, there was no significant change in the current required to reach 63% of the maximal firing frequency (from 0.55±0.06 nA (n = 14) under control conditions to 0.52±0.08 nA (n = 4) after application of 8 µM NMDA and 0.8 µM AMPA). Application of synaptic blockers significantly reduced this value to 0.46±0.04 nA (n = 8, p<0.01, paired t-test). Note that blocking synaptic transmission under control conditions in ACSF did not change the F-I at all (data not shown).

## Discussion

The experiments here were designed to investigate the working hypothesis that slice excitation media will induce changes to both cellular and network parameters. We examined three excitatory media, two with modified ionic composition and one with various concentrations of neurotransmitters added to the standard ACSF. Using blockers of synaptic transmission we show that modifying the ionic composition of the ACSF causes distinct changes in the cellular properties both at room temperature and at 35°C. Thus, the impact of slice excitation solutions on the physiology of single neurons may result from both cellular and network contributions. The ionic composition of the CSF measured *in vivo* suggests that the currently standard ACSF depresses network activity in acute brain slices, possibly influencing the outcome of experiments on the biophysical and physiological properties of neurons in acute brain slices. Our results suggest that it may be necessary to redesign experiments that have been carried out in a quiescent acute brain slice preparation.

Application of both ACSF_1_ and ACSF_2_ reduced neuronal R_in_ in the slice (Fig. 6 and Fig. S3). A trivial explanation is that the increase in network activity increases the rate of synaptic input to the neuron, reducing its R_in_. But this explanation must be rejected, as addition of synaptic blockers to both ACSF_1_ and ACSF_2_ did not reverse the change in R_in_ (Fig. 6 and Fig. S3). Thus, it appears that the reduction in R_in_ is due to the differential activation of ion channels in the standard ACSF and the excitatory media. This hypothesis is partially supported by the failure of ACSF_3_ to reduce R_in_ of L5 pyramidal neurons regardless of the concentrations of added neurotransmitters (Fig. 6C and Fig. S3C).

The reduction in R_in_ generated by both ACSF_1_ and ACSF_2_ is highly significant for the integrative properties of the neuron. Assuming that a reduction in R_in_ indicates a reduction in membrane resistance, then this change decreases the membrane time constant and the passive space constant. These changes, in turn, increase the filtration of synaptic input, further attenuating distal synaptic input as it travels to the soma. The reduction in R_in_ may explain the shift in the F-I curve to higher current values recorded in ACSF_2_ (Fig. 7). The shift in the F-I curve to lower current values in ACSF_1_ cannot be explained with the same logic and is probably a more complex effect combining modulation of the activity of ion channels.

Application of ACSF_1_ generated a significant hyperpolarization in the membrane potential at room temperature and depolarization at 35°C, while ACSF_2_ generated a small but insignificant depolarization (Figs 3, 4, and Fig. S2). The hyperpolarization induced by ACSF_1_ may be attributed to outward K^+^ current via inward rectifier K^+^ channels [30], which have been observed in the soma and dendrites of L5 pyramidal neurons [31], [32]. Although, it is clear from the opposite effect at higher temperature that more ion channels are probably responsible for the control of the resting membrane potential. It is harder to explain the depolarization induced by ACSF_2_, which could result from the activation of several ionic conductances at subthreshold potentials. ACSF_2_ also considerably increased the variance of the subthreshold membrane potential (Figs 3 & 4). Again, the trivial explanation is that this is due to an increase in the rate of synaptic input. However, this explanation must be rejected, because the membrane potential variance measured in ACSF_2_ did not fall back to control values following application of synaptic blockers (Figs 3 & 4).

Low concentrations of AMPA and NMDA generated only subthreshold activity in L5 pyramidal neurons (Fig. 4). This may be due to direct activation of glutamate receptors in the membrane of the neuron under investigation, since activation of the entire network would have generated action potential firing [21]. At higher neurotransmitter concentrations it is likely that both network and local channel activation are responsible for the effect of ACSF_3_. However, it is not possible to prove this hypothesis at present since it is not possible to block receptors only on the membrane of the neuron under investigation.

Increasing the temperature from room temperature (20–22°C) to 35°C decreased the spontaneous firing rate of L5 pyramidal neurons (Figs 2 and 3). One explanation of this effect may be that higher temperature increases the rate constants of channel gating thus speeding the processes of opening and closing of both voltage- and ligand-gated channels. The higher excitability of voltage-gated channels is manifested in the F-I curves that extend to higher frequencies for the same level of current injection (compare Fig. 7 to Fig. S3). Faster kinetics of AMPA and NMDA receptors will be manifested in shorter EPSPs. Thus, more EPSPs will be required to reach AP threshold leading to a lower spontaneous AP firing rate. Alternatively, the higher temperature shifted the excitation to inhibition ratio of the synaptic network in which L5 pyramidal neurons are embedded. While of importance to the future design of experiments in active brain slices the analysis of the firing rate did not assist dissecting between the cellular and network aspect of slice excitation by the various ACSF solutions.

The experiments here clearly show that small changes in the extracellular [K^+^] and [Ca^2+^] may cause large changes in the level of excitability of L5 pyramidal neurons and of the surrounding neuronal networks. Therefore, which of the tested solutions is closest to *in vivo* concentrations? Extracellular [K^+^] and [Ca^2+^] have been repeatedly measured (for review see [33]). At rest [Ca^2+^] ranges between 1 and 1.5 mM and [K^+^] ranges between 3 and 3.5 mM. During trauma [K^+^] can increase substantially, while [Ca^2+^] may decrease [33]. Thus, ACSF_1_ possibly mimics *in vivo* concentrations better than the other solutions tested here, while ACSF_2_ appears to mimic the pathological extracellular ionic combination following trauma or during an epileptic seizure [33]. Having said that, it is important to mention that the major problem of the brain slice preparation is the absence of sensory and other inputs it receives *in vivo*. Thus, the activity of the neurons cannot in our opinion be directly compared to *in vivo* activity. It is possible to use the brain slice preparation to investigate the function of single neurons and small neuronal networks. However, we would be very hesitant to extrapolate our findings to the *in vivo* situation.

Most if not all investigations of long-term changes in synaptic efficacy employ complex pairing protocols between pre- and post-synaptic neurons relying on precise timing between the firing of the two neurons in the circuit [3], [34]. In excited slice preparations, action potentials arriving in a seemingly random fashion from the network may have a large positive or negative impact on the performance of the commonly used pairing protocols. Dendritic synaptic integration may also be greatly affected by the ionic composition of the medium. Action potentials initiated at or near the soma actively back-propagate into the dendritic tree [2], [35]–[39]. The dendrites generate complex regenerative Ca^2+^ and Na^+^ spikes [35], [40]–[49] and modulate synaptic potentials [50], [51]. Modulation of the extracellular calcium concentration may profoundly affect the initiation and propagation of dendritic calcium spikes, as has been recently demonstrated in pyramidal neurons of the CA1 region of the hippocampus [52].

In conclusion, any investigation of cellular or synaptic plasticity in the quiescent brain slice preparation should bear in mind that many cellular and network properties may deviate considerably from those *in vivo* conditions. The study we presented here clearly indicates that any such investigation requires more controls and more attention to the relevance of the experimental protocols to realistic conditions.

## Supporting Information

Figure S1 Data analysis procedures
(0.03 MB PDF)

Figure S2 Effect of slice excitation on average membrane potential, variance, action potential amplitude and threshold recorded at 35°C
(0.04 MB PDF)

Figure S3 Modified ionic composition of ACSF changes input resistance of L5 pyramidal neurons and its response to injected current recorded at 35°C
(0.06 MB PDF)
