# Supplementary material for: Cellular and Network Contributions to Excitability of Layer 5 Neocortical Pyramidal Neurons in the Rat
Source: PLoS One. 2007 Nov 21;2(11):e1209. doi: 10.1371/journal.pone.0001209 (PMC2075161; doi:10.1371/journal.pone.0001209)
Supplement: Figure S1 — Data analysis procedures (0.03 MB PDF) [file pone.0001209.s001.pdf]

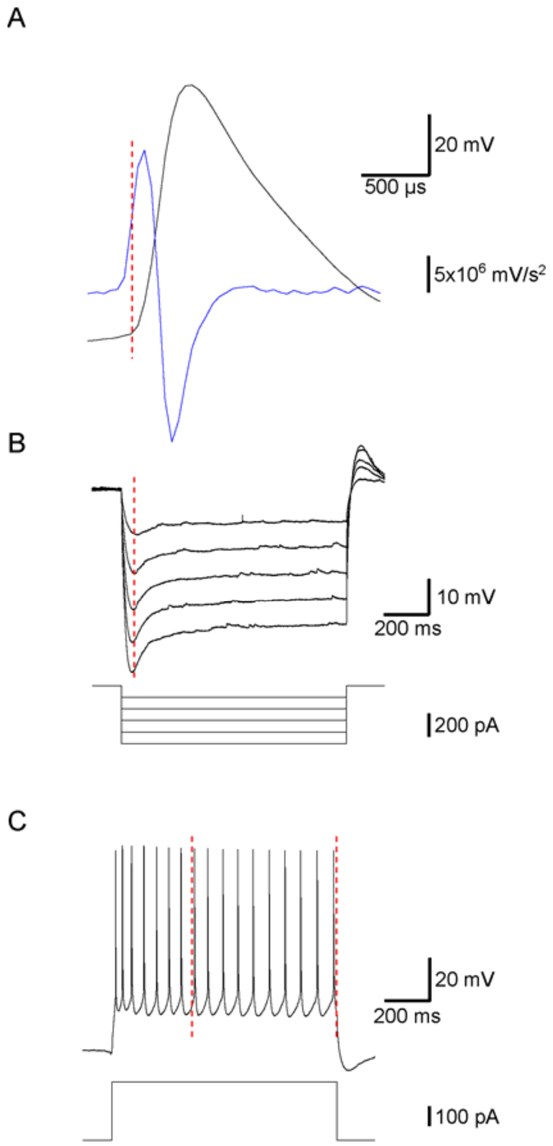

Figure S1: Data analysis procedures. A, graphical demonstration of the numerical procedure used to calculate AP threshold. The membrane potential during the action potential (black line) is displayed with its numerical second derivative (blue line). A vertical red line indicates the 50 % change in the second derivative and the location of the AP threshold on the membrane potential trace. B, graphical demonstration of the numerical procedure used to calculate input resistance. Membrane potential traces recorded in response to negative current injection via the patch pipette. A vertical red line indicates the values used to calculate the input resistance before the passive response was contaminated by the activation of I_h. C, graphical demonstration of the numerical procedure used to calculate the mean firing frequency of the neuron following positive current injection. In all traces the first 400 ms were discarded and the analysis was performed on the following 600. The red lines indicate the section of the data used for analysis.
