# Supplementary material for: Cellular and Network Contributions to Excitability of Layer 5 Neocortical Pyramidal Neurons in the Rat
Source: PLoS One. 2007 Nov 21;2(11):e1209. doi: 10.1371/journal.pone.0001209 (PMC2075161; doi:10.1371/journal.pone.0001209)
Supplement: Figure S2 — Effect of slice excitation on average membrane potential, variance, action potential amplitude and threshold recorded at 35°C (0.04 MB PDF) [file pone.0001209.s002.pdf]

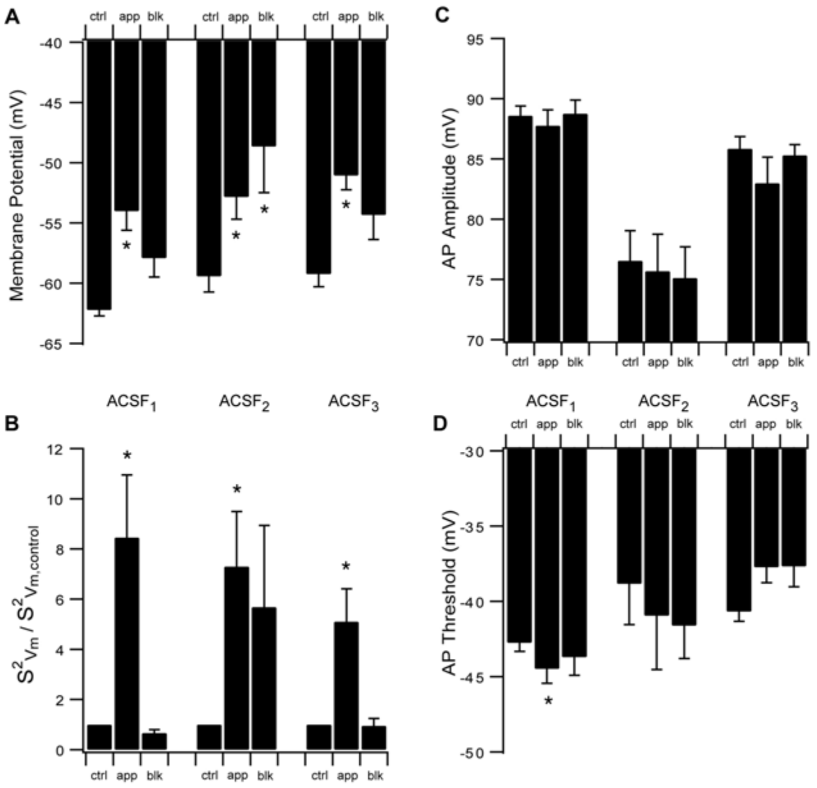

Figure S2: Effect of slice excitation on average membrane potential, variance, action potential amplitude and threshold recorded at 35ºC. Membrane potential, membrane potential variance, AP amplitude and AP threshold were measured under control conditions, application of modified ACSF and addition of synaptic blockers. A, average membrane potential with $ACSF_1$ (left) which depolarized significantly during application (n=4, $p<0.01$ paired t-test), $ACSF_2$ (middle) that depolarized significantly following application (n=5, $p<0.05$ paired t-test) and blockers (n=5, $p<0.05$ paired t-test), and $ACSF_3$ with 8 µM NMDA and 0.8 µM AMPA (right) which also presented significant depolarization during application (n=6, $p<0.005$ paired t-test). B, normalized membrane potential variance with $ACSF_1$ (left), $ACSF_2$ (middle) and $ACSF_3$ with 8 µM NMDA and 0.8 µM AMPA (right). In all 3 solutions a considerable increase occurred during application ($ACSF_1$: n=6, $p<0.05$ paired t-test; $ACSF_2$: n=7, $p<0.05$ paired t-test; $ACSF_3$: n=6, $p<0.05$ paired t-test). C, AP amplitude with $ACSF_1$ (left), $ACSF_2$ (middle) and $ACSF_3$ with 8 µM NMDA and 0.8 µM AMPA (right). D, AP threshold with $ACSF_1$ (left) displaying statistically significant decrease from -42.7±0.56 mV in control to -44.5±0.95 mV in application (n=6, $p<0.05$ paired t-test), $ACSF_2$ (middle) and $ACSF_3$ with 8 µM NMDA and 0.8 µM AMPA (right).
