# Supplementary material for: Cellular and Network Contributions to Excitability of Layer 5 Neocortical Pyramidal Neurons in the Rat
Source: PLoS One. 2007 Nov 21;2(11):e1209. doi: 10.1371/journal.pone.0001209 (PMC2075161; doi:10.1371/journal.pone.0001209)
Supplement: Figure S3 — Modified ionic composition of ACSF changes input resistance of L5 pyramidal neurons and its response to injected current recorded at 35°C (0.06 MB PDF) [file pone.0001209.s003.pdf]

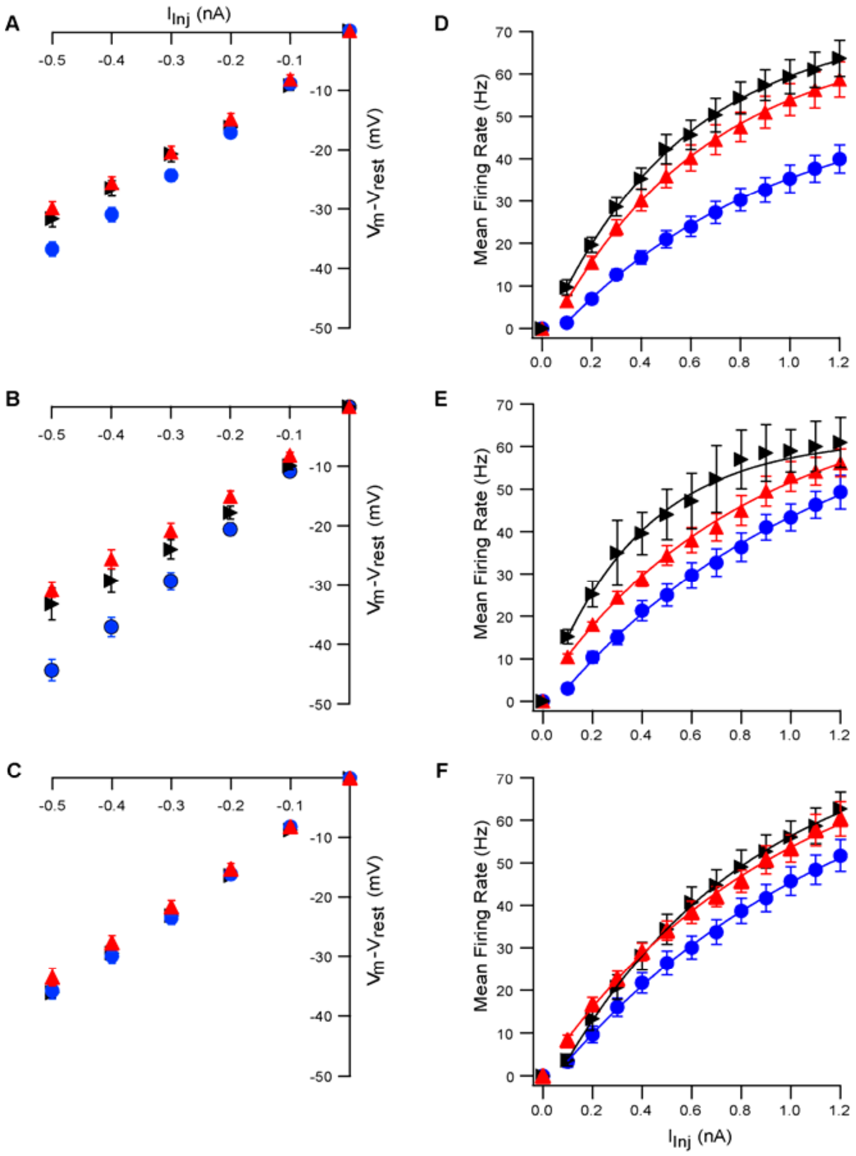

Figure S3: Modified ionic composition of ACSF changes input resistance of L5 pyramidal neurons and its response to injected current recorded at 35ºC. A, B, and C data taken in the control condition (blue circles), application of modified ACSF (red upward triangles) and application of modified ACSF with synaptic blockers (black rightward triangles). A, voltage-current curves with $ACSF_1$. Input resistance was 73.46±2.47 MΩ in control and decrease to 59.27±2.005 MΩ (n=6, p<0.001 paired t-test) and did not retrieve with blockers (62.25±2.49 MΩ, n=6, p<0.001 paired t-test). B, voltage-current curves with $ACSF_2$. Same trend was observed as with $ACSF_1$ (control: 88.31±3.5 MΩ; application: 59.06±3.29 MΩ; blockers: 61.4±3.53 MΩ; control-application: n=7, p<0.001 paired t-test; control-blockers: n=6, p<0.005 paired t-test). C, voltage-current curves with $ACSF_3$ with 8 µM NMDA and 0.8 µM AMPA. Similar curves observed along control (71.88±2.84 MΩ), application (68.2±2.78 MΩ) and blockers (71.29±1.66 MΩ). Response to injected current was measured and plotted by exponential fitting to curves from AP frequency vs. injected current. D, E and F, control (blue circles), application of modified ACSF (red upward triangles) and addition of synaptic blockers (black rightward triangles). D, $ACSF_1$; under control conditions the current required to induce a firing rate of 63 % of the maximal firing rate was 0.95±0.049 nA and shifted to lower current value of 0.66±0.022 nA (n=6, p<0.0001 paired t-test) and remained lower with blockers (0.55±0.019 nA, n=6, p<0.0001 paired t-test). E, $ACSF_2$; the current required to induce a firing rate of 63 % of the maximal firing rate was 1.18±0.1 nA. Application constructed current values of 1.15±0.19 nA (n=6, p<0.0001 paired t-test), and with blockers those values shifted to lower currents (0.47±0.102 nA, n=5, p<0.001 paired t-test). F, $ACSF_3$ with 8 µM NMDA and 0.8 µM AMPA; during control 1.27±0.102 nA (n=6) was enough to produce 63 % of the maximal firing rate, decreased to 1.13±0.105 nA (n=6) and continued to 0.804±0.032 nA (n=6) with blockers.
